# Saffron administration prevents selenite-induced cataractogenesis

**Published:** 2013-05-30

**Authors:** Olga E. Makri, Anastasia-Varvara Ferlemi, Fotini N. Lamari, Constantine D. Georgakopoulos

**Affiliations:** 1 Department of Ophthalmology, Medical School, University of Patras, Patras, Greece; 2 Laboratory of Pharmacognosy & Chemistry of Natural Products, Department of Pharmacy, University of Patras, Patras, Greece

## Abstract

**Purpose:**

The present study sought to investigate whether *Crocus sativus* stigmas (saffron) extract prevents selenium-induced cataractogenesis in vivo, and to study its possible protective mechanism.

**Methods:**

Wistar rat pups were randomized into three groups. Group I (control) received subcutaneous injection of normal saline on postnatal day 10. Groups II (selenite-treated) and III (selenite+saffron-treated) received subcutaneous injection of sodium selenite (20 µmol/kg body weight) on postnatal day 10. Group III also received intraperitoneal injections of saffron extract (60 mg/kg body weight) on postnatal days 9 and 12. On postpartum day 21, rats were sacrificed and the lenses were isolated and examined for cataract formation. Activities of superoxide dismutase, glutathione peroxidase, catalase, and glutathione levels, as markers of antioxidant defense, were measured in the isolated lenses. Levels of the indicator of lipid peroxidation, malondialdehyde, and protein oxidation (sulfhydryl content) in the lens were also determined. The effect of the different treatments on lens protein profile was evaluated through an estimation of the soluble to insoluble protein ratio and sodium dodecyl sulfate polyacrylamide gel electrophoresis analysis of the water-soluble fraction (WSF) of lens proteins.

**Results:**

Saffron demonstrated significant protection against selenite-induced cataractogenesis in vivo. The mean activities of superoxide dismutase, glutathione peroxidase, catalase, and glutathione levels were significantly increased in group III compared to the selenite-treated group. Saffron significantly prevented selenite-induced lipid peroxidation, protein oxidation, and proteolysis and insolubilization of the lens WSF.

**Conclusions:**

Saffron extract prevented selenite-induced cataract formation in Wistar rats, possibly through the reinforcement of antioxidant status, reduction of the intensity of lipid peroxidation, protection of the sulfhydryl groups, and inhibition of proteolysis of the lens WSF. These findings highlight the anticataractogenic potential of saffron by virtue of its antioxidant property.

## Introduction

Cataract is a degenerative condition and results from the loss of crystalline lens transparency. According to data provided by the World Health Organization, cataract is one of the major causes of visual impairment globally and the first cause of blindness, accounting for 51% of cases worldwide [1]. It is estimated that about 40 million people will have severely reduced vision due to cataract by the year 2020 [2], especially in the developing countries of Asia and Africa [3]. The only available treatment for cataract is the surgical removal of the opaque lens and its replacement with an artificial one to restore visual acuity. The aforementioned procedure has been optimized over the last decades so as to become an everyday surgery in developed countries. Nevertheless, cataract-induced blindness remains a severe health problem in developing countries due to the low socioeconomic status of the patients and limitations in the acceptability, accessibility, and affordability of cataract surgical services [4].

Since surgical treatment is not widely available, many researchers have investigated pharmaceutical agents to prevent this disease. It has been estimated that a delay of 10 years in the onset of cataract, by any means, would halve the number of patients needing surgery [2]. Actually, over the last few years, great emphasis has been placed on exploring the possible protective effects of several natural products against cataract formation. Natural products are privileged candidates for drug discovery, since they are often endowed with multiple functions. Cataractogenesis is considered to be a multifactorial disease, correlated with various pathogenetic mechanisms that have not been completely clarified. There is a large body of evidence demonstrating that oxidative stress (i.e., measurably increased levels of reactive oxygen species and oxidized substrate molecules—lipids, sulfhydryls, nucleic acids) is compulsory to cataract development [5-9]. Consequently, enhancement of the antioxidant defenses of the lens could prevent or delay the onset of cataract. Based on that premise, numerous natural products with known antioxidant properties have been evaluated in the last decades.

Saffron consists of the dried stigmas of *Crocus sativus* L., which is a bulbous perennial plant of the family *Iridaceae*. Saffron has been used and consumed since antiquity, and is one of the most expensive spices; it is used for flavoring and coloring food preparations, as a perfume, and also as a dye. It is cultivated in many Mediterranean countries, including Spain, Greece, and Turkey, as well as in India and Iran (major producer) [10]. The value of saffron is attributed to its characteristic phytochemical components, specifically the unique hydrophilic carotenoids, i.e., crocins (glycosidic esters of crocetin), picrocrocin (terpenoid glycoside responsible for the exquisite taste), and safranal (the main essential oil component) [11-13]. Hippocrates and Dioscurides mention the use of saffron for the treatment of ophthalmic disorders, among other uses. Since then, saffron has been constantly used in traditional medicine, as therapy for several conditions such as insomnia, depression, bronchospasm, cardiovascular diseases, gastrointestinal disorders, menstrual pain, menopausal problems, as analgesic, and even against cancer [10,14]. Some of the medicinal-biologic properties of saffron or its components are attributed to its antioxidant features, which have been highlighted in several studies [15-21]. The objective of the present study is to investigate, for the first time in the literature, the effect of saffron extract against selenite-induced experimental cataract in vivo.

## Methods

### Chemicals

Commercially available saffron was kindly provided by the Cooperative de Safran (Krokos Kozanis), Krokos, West Macedonia, Greece. Sodium selenite (44%–46%), catalase (CAT) from bovine liver (4966 U/mg), Purpald, N-ethylmaleimide, reduced L-glutahione, oxidized glutathione form, glutathione peroxidase (GPx), and N-acetyl-cysteine were purchased from Sigma-Aldrich (St. Louis, MO). A Superoxide Dismutase Assay Kit from Cayman Chemical Company (Ann Arbor, USA) was used for the determination of superoxide dismutase (SOD) activity.

### Preparation of saffron extract

Saffron stigma powder was extracted with methanol:water (50%v/v; 18 ml/250 mg) for 4 h at room temperature, in the absence of light and with continuous stirring. The extract was centrifuged, filtered, and evaporated to dryness using a Speed Vac System (Labconco Corp., Kansas City, MO). The composition was screened with high-performance liquid chromatography (HPLC) on a Supelcosil C-18 column with a gradient of methanol containing 1% v/v acetic from 20 to 70% in 50 min [13,20].The residues were stored at −20 °C until further use. Samples were redissolved in normal saline and sterilized though membrane filtration (0.2 μm i.d.) for the injections.

### Animals

The experiments were conducted in strict compliance with the Association for Research in Vision and Ophthalmology Statement for Use of Animals in Ophthalmic and Vision Research and Guiding Principles in the Care and Use of Animals [22]. The study has been approved by the University Hospital Bioethics Committee.

Newborn suckling Wistar rat pups were used in our experiment. The pups were housed along with their mothers in stainless-steel cages in well-ventilated rooms with controlled temperature (23 °C) and humidity conditions and 12 h:12 h light-dark cycles. The mothers were maintained on a standard laboratory animal diet in the form of dry pellets and provided tap water ad libitum throughout the experimental period.

Animals were randomly assigned to three groups:

• Group I (control, n=9 animals), which received only subcutaneous injection of normal saline;

• Group II (selenite-treated, n=8 animals), which received a subcutaneous injection of sodium selenite (20 µmol/kg body weight) on postnatal day 10; and

• Group III (selenite+saffron-treated, n=9 animals), which received a subcutaneous injection of sodium selenite (20 µmol/kg body weight) on postnatal day 10 and intraperitoneal injections of saffron extract (60 mg/kg body weight) on postnatal days 9 and 12.

Cataract formation could be evaluated from the 16th day, when the pups opened their eyes, with the help of an ophthalmoscope and later on with the naked eye. Bilateral cataracts were observed in the animals used for this study. On postpartum day 21, rats were anaesthetized by placing them in a bell jar containing cotton wool soaked in diethyl ether. The animals were kept inside the closed jar until loss of righting reflex, and then sacrificed by cervical dislocation. The eyes were enucleated and lenses were at once excised intracapsularly through an incision 2 mm posterior to the limbus under surgical microscope magnification. Both lenses were obtained from each rat and morphological examination for cataract formation was performed by gross examination of lenses under the magnification of the dissecting microscope against a background of black gridlines.

**Figure 1:**
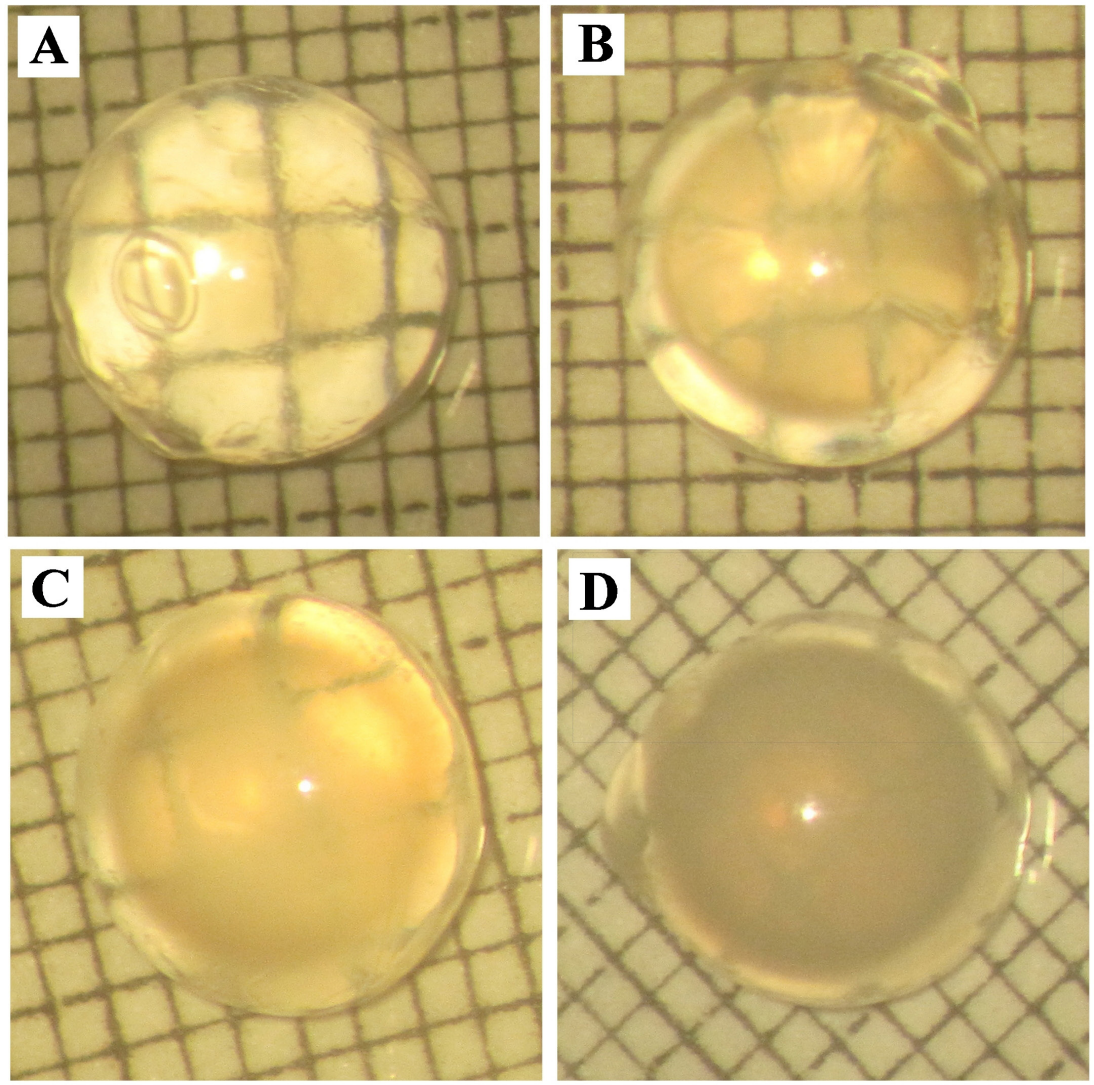
Transillumination pictures of lenses representative of each grade. Lens opacification of experimental animals was assessed to a specific grade in the range of 0 to 3. Each picture (**A**-**D**) is representative of each grade. **A**: Grade 0: absence of opacification (gridlines clearly visible); **B**: Grade 1: a slight degree of opacification, with minimal clouding of gridlines; **C**: Grade 2: diffuse opacification involving almost the entire lens, with gridlines faintly visible; **D**: Grade 3: extensive dense opacification involving the entire lens (gridlines not visible).

Staging of the cataract formation was conducted by an examiner, blinded regarding the study groups, based on a scale 0 to 3 from Geraldine et al. [23]. The degree of opacification was graded as follows:

• Grade 0: absence of opacification (gridlines clearly visible; Figure 1A);

• Grade 1: a slight degree of opacification (minimal clouding of gridlines, with gridlines still visible; Figure 1B);

• Grade 2: presence of diffuse opacification involving almost the entire lens (moderate clouding of gridlines, with gridlines faintly visible; Figure 1C);

• Grade 3: presence of extensive thick opacification involving the entire lens (total clouding of gridlines, with gridlines not seen at all; Figure 1D)

### Tissue treatment

Prior to biochemical analysis, the lenses were washed in ice-cold saline to remove blood and then weighed carefully. The lenses were homogenized in 30 mM phosphate buffer, pH 7.6 (10% w/v), and centrifuged at 12,600 ×g for 20 min at 4 °C. The supernatant obtained was stored at −30 °C, pending further analysis.

### Analytical methods

#### Assay of superoxide dismutase activity

SOD activity was determined according to the Superoxide Dismutase Assay Kit, Cayman Chemical Company, which is based on the formation of a tetrazolium salt for the detection of superoxide radicals generated by xanthine oxidase and hypoxanthine. The absorbance was read at 450 nm. The results are expressed as units/ml, where one unit of SOD is defined as the amount of enzyme needed to exhibit 50% dismutation of the superoxide anion.

#### Assay of catalase activity

The peroxidative activity of CAT was determined according to the modified method of Sinha [24]. The method is based on the reaction of the enzyme with methanol in the presence of an optimal concentration of H_2_O_2_. The formaldehyde produced is measured colorimetrically at 540 nm with reaction with Purpald (4-amino-3-hydrozino-5-mercapto-1,2,4-triazole) and oxidation with KIO_4_. Standard concentrations of formaldehyde (0–75 μΜ) were used for the construction of a calibration curve. All determinations were performed in triplicate. The results are expressed as nmol/min/ml.

#### Glutathione peroxidase activity

GPx activity was determined colorimetrically following Rotruck et al. [25] to estimate the rate of the glutathione oxidation by H_2_O_2_. Reduced glutathione was used as substrate and 5,5′-dithiobis(2-nitrobenzoic acid) as a chromogen. Suitable aliquots of standard concentrations of GPx (0.025–1 U/ml) were also used for the construction of the calibration curve. The color that was developed was read against a reagent blank at 412 nm. Results were expressed as units/mg tissue (one unit was the amount of enzyme that converted 1 μmol of reduced to the oxidized form of glutathione in the presence of H_2_O_2_/min).

### Estimation of reduced and total glutathione content

A modification of Hissin and Hilf’s method was used for the determination of reduced (GSH) and oxidized glutathione (GSSG) content in the lens [26]. The two forms of glutathione were estimated fluorometrically after reaction with *o*-phthalaldehyde (OPT). The samples were treated with 50% trichloroacetic acid (TCA) and centrifuged to eliminate the protein content. The supernatant was extracted three times with diethyl ether. The aqueous fraction was used to determine the GSH and GSSG content; GSH selectively reacted with OPT (10 mg/ml in pure methanol) at pH 8.0 (500 mM Na_2_HPO_4_ buffer), whereas after the addition of 5 mM N-ethyl maleimide (NEM) in the samples, only GSSG reacted with OPT at pH 12.0 (0.2 N NaOH). Standard concentrations of GSH and GSSG (0.75–15 μΜ) were used. The excitation wavelength was at 340 nm and the fluorescence intensity was determined at 420 nm. The respective concentrations in nmol/mg wet tissue were calculated from the respective calibration curves. The concentration of total glutathione was calculated by adding GSH and GSSG.

### Estimation of protein sulfhydryl content

The protein sulfhydryl content was estimated using Ellman’s procedure as slightly modified by Sedlak and Lindsay [27]. Briefly, the pellets from the samples treated with 50% TCA for the determination of glutathione were redissolved with a Tris-EDTA-guanidine HCl (3 mM: 3 mM: 3M) buffer, pH 8.9. In a 96-well microplate, 250 μl of the sample was mixed with 20 μl 5,5′-dithiobis(2-nitrobenzoic acid). After incubation for 15 min at room temperature, the absorbance was read at 412 nm. The content of protein sulfhydryls was calculated using a calibration curve prepared with *N*-acetyl-cysteine (0.06 mM–1 mM). The protein content of each sample was evaluated using the Bradford assay [28]. Since N-acetyl-L-cysteine (NAC) contains only one sulfhydryl group, the results were expressed as nmol –sulfhydryl group (SH)/mg protein.

### Determination of lipid peroxidation

Lipid peroxidation was determined by the evaluation of malondialdehyde (MDA) levels in the rat lens. Due to the small volume of tissue sample, the fluorimetric method of Grotto et al. [29] and Jentzsch et al. [30] was used with slight modifications to measure MDA levels using a 96-well microplate. MDA was determined after the reaction with thiobarbituric acid and extraction with *n*-butanol. The excitation wavelength was at 515 nm and the fluorescence intensity was determined at 553 nm. The results were expressed as nmol MDA/g tissue against a standard curve (0.05–10 μΜ MDA).

### Sodium dodecyl sulfate polyacrylamide gel electrophoresis of water soluble proteins

Samples (30 μg protein) were loaded on a 12% polyacrylamide gel (1 mm thickness) according to a modification of Laemmli’s method [31]. Before loading, lens homogenate was mixed with sample buffer (35% v/v glycerol, 18% v/v 2-mercaptoethanol, 230 mM Tris/HCl pH=6.8, 10% w/v sodium dodecyl sulfate [SDS], 0.3% w/v bromophenol blue). Each mixture was heated at 100 °C for 5 min and then centrifuged for 3 min. The protein mix was left to cool at room temperature. Electrophoresis was performed using a Cleaver Scientific Ltd (Warwickshire, Rugby, UK) apparatus at 100V for approximately 2 h. For the visualization of protein lanes, gels were stained with 0.5% w/v Coomassie brilliant blue in methanol: acetic acid: water (3:1:6 v/v/v) and destained. Image J Launcher was used for the densitometric analysis of gels (at least four different experiments).

### Determination of lens proteins

The determination of insoluble and soluble protein content in the rat lenses of each group was estimated using the Bradford assay, with bovine serum albumin as a standard [28]; the results were expressed as mg protein/ mg wet tissue. The ratio of soluble to insoluble proteins per sample was calculated.

### Statistical analysis

All variables were tested for normality with the Kolmogorov–Smirnov test. Differences in clinical score between the groups were estimated with the Mann–Whitney *U* test. One-way analysis of variance was used to detect differences in continuous variables among the three groups. The Bonferroni post hoc test was used for pairwise comparisons. A p value less than 0.05 was considered statistically significant. The analysis was performed using SPSS 15.0 software (SPSS, Inc., Chicago, IL).

## Results

### Morphological assessment of cataract formation

In group I, all the eyes were clear. In group II, 100% of the eyes developed moderate to severe cataract, indicating success in establishing the selenite-induced cataract model in our experiment. Stage 2 was the common endpoint of the cataractogenic process, accounting for 62.5% of the eyes (median 2, range 2–3). In contrast, saffron coadministration (group III) significantly retarded selenite-induced cataractogenesis in vivo. In this group, 38.9% of the lenses exhibited stage 1 cataract, 44.4% of the eyes developed stage 2 cataract, and only 16.7% developed dense cataract (median 2, range 1–3; Table 1, Figure 1).The difference in cataract exhibited in the control and selenite-treated group was significant (p<0.0001, Mann–Whitney *U* test). The difference in cataract frequency and severity between groups II and III was statistically significant (p<0.05, Mann–Whitney *U* test).

**Table 1:** Morphological assessment of cataract formation of each group’s isolated lenses

Group	No of lenses with different degree of opacification				
	Grade 0	Grade 1	Grade 2	Grade 3	Median (range)
I (n=18)	18	0	0	0	0 (0)
II (n=16)	0	0	10	6	2 (2–3) *
III (n=18)	0	7	8	3	2 (1–3) ^*, ‡^

Incidence and staging of cataract formation of the isolated lenses in each experimental group. Group I: Control, Group II: Selenite-treated, Group III: Selenite+Saffron-treated. The Mann–Whitney *U* test was used for pairwise comparisons. Different symbols indicate statistically significant difference between groups. * p<0.0001 compared with group I. ^‡^ p<0.05 compared with group II.

### Antioxidant status in the lens

#### Superoxide dismutase activity

The activity of SOD (mean±standard deviation [SD]) was significantly lower in the lenses of group II compared to that of normal lenses (p<0.001; Table 2). However, saffron coadministration (group III) resulted in significantly higher SOD activity levels compared to group II, which received only sodium selenite (p<0.05), although they were lower than those of the group I (control; p<0.05).

**Table 2:** Activities of antioxidant enzymes of the lenses of each group.

Enzyme	Group I	Group II	Group III
Superoxide dismutase (unit/ml)	0.37±0.07	0.18±0.05**	0.26±0.03^#, ‡^
Catalase (nmol/min/ml)	0.07±0.01	0.04±0.005*	0.067±0.02^‡^
Glutathione peroxidase (μmol glutathione oxidized/min/mg tissue)	14.71±1.39	7.39±1.30**	12.34±1.93^†^

Activities of the enzymes superoxide dismutase, catalase and glutathione peroxidase of the lenses of each experimental group. Group I: Control, Group II: Selenite-treated, Group III: Selenite+Saffron-treated. Each value represents the mean ± SD of six determinations. Statistical analysis was performed by one-way ANOVA with Bonferroni’s adjustment . Different symbols indicate statistically significant difference between groups of each enzyme activity. *p<0.01; **p<0.001; ^#^p<0.05 compared with group I. ^‡^p<0.05; ^†^p<0.001 compared with group II.

### Catalase activity

The mean activity of CAT (mean±SD) in the lenses of group II rats was significantly lower than that of the lenses in control group (p<0.01; Table 2). In group III, CAT activity was significantly higher than in group II (p<0.05). In fact, saffron coadministration resulted in the prevention of the loss of CAT activity, which was measured at levels similar to those of the control group (p>0.05).

### Glutathione peroxidase activity

The mean activity of GPx (mean±SD) in lenses of group II was significantly lower than that of the lenses in the control group (p<0.001; Table 2). In the selenite+saffron treated group (III), GPx activity was significantly higher than in the selenite-treated group (p<0.001).

### Reduced and total glutathione

For the further evaluation of the antioxidant status of the lenses, the reduced and total glutathione levels (mean±SD) were evaluated (Table 3). Selenite administration resulted in a significant reduction of reduced glutathione in comparison with normal lenses (p<0.05). Cotreatment with saffron (group III) restored the reduced glutathione concentration, which reached a level that was not statistically significantly different from the control group (p>0.05). The lens total glutathione pool was depleted after selenite administration, while saffron showed a sparing effect on total glutathione content.

**Table 3:** Total and reduced glutathione levels.

Parameters	Group I	Group II	Group III
Reduced glutathione (nmol/mg wet tissue)	0.33±0.1	0.17±0.02*	0.31±0.13
Total glutathione (nmol/mg wet tissue)	0.50±0.17	0.36±0.07	0.52±0.18

Reduced and total glutathione levels in the lens of Wistar rat pups of each group. Group I: Control, Group II: Selenite-treated, Group III: Selenite+Saffron-treated. Values represent the mean ± SD of six determinations. Statistical analysis was performed by one-way ANOVA with Bonferroni’s adjustment. Symbol indicates statistically significant difference between groups II and I (p<0.05)

### Levels of lens protein sulfhydryls

The level of protein sulfhydryl groups is an important indicator of tissue protein oxidation. In group II, protein sulfhydryl content (mean±SD) was significantly lower compared to control (p<0.001; Table 4). However, in group III, lens protein sulfhydryl content was significantly higher than the concentration observed in the selenite-treated group (p<0.05), demonstrating its protective effect against protein oxidative damage. Sulfhydryl content in group III reached a level that was not statistically significant lower than group I (p>0.05).

**Table 4:** Levels of the indicators of protein (sulfhydryl content) and lipid (malondialdehyde) oxidation and ratio of soluble/insoluble proteins in lens of each group.

Component analyzed	Group I	Group II	Group III
Protein sulfhydryl (nmol -SH/mg protein)	0.93±0.1	0.67±0.08**	0.82±0.1^‡^
Malondialdehyde (nmol/g tissue)	56.64±12.38	108.51±13.63*	49.04±18.22^†^
Soluble/insoluble proteins	2.91±0.09	1.88±0.27*	2.28±0.27**^, ‡^

Quantitative analysis of protein sulfhydryl groups and malondialdehyde levels in the lenses of each experimental group. Calculated soluble to insoluble proteins ratio in each group lenses. Group I: Control, Group II: Selenite-treated, Group III: Selenite+Saffron-treated. Data represent the mean ± SD of six determinations. Statistical analysis was performed by one-way ANOVA with Bonferroni’s adjustment. Different symbols indicate statistically significant difference between groups. *p<0.0001; **p<0.001 compared with group I. ^†^p<0.0001; ^‡^p<0.05 compared with group II.

### Levels of the indicator of lipid peroxidation in lens (malondialdehyde)

The MDA level (mean±SD) reflects the overall tissue lipid peroxidation [32]. In group II, this was 91.6% higher than in group I (p<0.0001; Table 4). In group III, it was 54.8% lower compared to the selenite-treated group (p<0.0001) and similar to that of the control group (p>0.05).

### Effect of saffron on lens proteins

Calculation of the soluble to insoluble ratio (mean±SD) revealed a significantly lower ratio in the selenite-treated group compared to the control group (p<0.0001; Table 4). In group III, saffron resulted in a significantly higher ratio in comparison to group II (p<0.05). The ratio in group III was still significantly lower than that of normal lenses (p<0.001).

**Figure 2:**
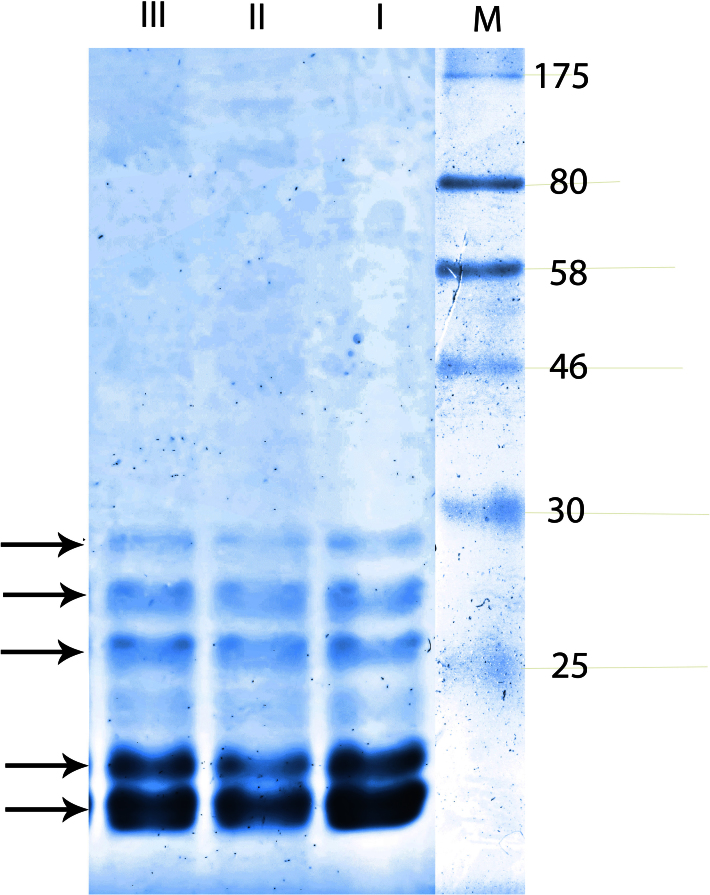
Sodium dodecyl sulfate polyacrylamide gel electrophoresis of the lens water soluble protein fraction. An equal amount of protein (30 μg) was loaded on 12% sodium dodecyl sulfate polyacrylamide gel electrophoresis (SDS–PAGE). The first right lane indicates the molecular weight marker; Group I lane represents the protein profile of the normal untreated lens; Group II, the selenite-treated group; Group III, the selenite+saffron-treated group. In the normal untreated lens water-soluble fraction (WSF), the prevalence of proteins with a molecular mass of 19–30 kDa (characteristic of crystallins) is shown. Selenite treatment led to a significant reduction (~35%) in these proteins, which was prevented by saffron coadministration.

The relative changes in the water-soluble fraction (WSF) of lens proteins were detected by SDS–polyacrylamide gel electrophoresis (PAGE) analysis. Figure 2 shows a significant decrease in low molecular weight proteins in the selenite-treated lens; proteins between 10 and 30 kDa in group II were lower (~35%) than those in group I. Saffron coadministration seems to diminish the observed proteolysis of these proteins, since the densitometric analysis revealed a significant increase of protein (25–30 kDa) band intensity (13%–27%) compared to group II. Particularly, the intensity of the 19–21 kDa protein band in group III was similar to that of the control (group I).

## Discussion

In recent years, studies have been conducted to evaluate the possible beneficial effects of saffron or its components in ophthalmology. All the published data make reference to the pathology of the retina and its possible beneficial effects in conditions such as ischemic retinopathy, age-related macular degeneration, or other neurodegenerative conditions [33-38]. However, despite the continuously increasing literature on the neuroprotective effects of saffron or its components in the retina, to date, no study has been conducted to evaluate its possible effects against cataract formation.

The purpose of our study was to examine in vivo the potential anticataract effects of saffron extract in selenite-induced cataract. Our clinical observations of the morphological examination of the lenses showed that cataract formation caused by selenite administration was attenuated by the administration of saffron, suggesting its anticataract potential. Furthermore, biochemical analyses were conducted to support the clinical observation.

The selenite cataract model was selected for its rapid, effective, and reproducible cataract formation [39,40]. Although the rate of lens opacification is much more rapid than in human cataract, the selenite model shows general similarities to human senile nuclear cataract, such as the formation of vesicles, increased levels of calcium, proteolysis, a decrease in water-soluble proteins, the presence of insoluble proteins, and diminished amounts of GSH. The mode of action of sodium selenite in cataractogenesis has not been completely defined, but it is indicated that the main biochemical events—induced by selenite overdose—are triggered by selenite-induced oxidative stress and the generation of reactive oxygen species in the aqueous humor in combination with a decrease in the activity of antioxidant enzymes such as SOD, CAT, GPx, glutathione transferase, and glutathione reductase, as well as GSH content in the lens [40,41]. More precisely, it is assumed that oxidative damage occurs to critical sulfhydryl groups of proteins of the lens epithelium membranes. This, in combination with oxidative stress–induced lipid peroxidative damage of lenticular membranes, leads to the inactivation of membrane proteins such as Ca^2+^-ATPase. Inhibition of the function of Ca^2+^-ATPase results in loss of calcium homeostasis and calcium accumulation in the lens. As a consequence of calcium influx in rodent lenses, calpains, a family of well-characterized calcium dependent proteases, are activated. Calpain activation, mainly m-Calpain and Lp82 in rodent lenses, induces rapid proteolysis of the water soluble proteins, that is, lens crystallins, and cytoskeletal proteins. Proteolysis exposes the hydrophobic regions of lens proteins, which then interact to form insoluble aggregates [39]. Insolubilization results in precipitation and aggregation of lens fragmented proteins, and finally, in loss of lens transparency [40].

SOD and CAT make up a primary line of defense against superoxide anion (O_2_^−^) and hydrogen peroxide (H_2_O_2_), respectively. More precisely, SOD is a specific scavenger of superoxide anion. It converts harmful superoxide radicals to H_2_O_2_, which is detoxified by CAT to harmless byproducts. GPx, which belongs to the family of selenoproteins, is present in relatively large amounts in the epithelium of the lens [42]. It catalyzes the reduction of a variety of hydroperoxides with the help of its reducing substrate, GSH. The enzyme GPx maintains the integrity of the phospholipid bilayer of membranes by putting a brake on the lipid peroxidation initiated by superoxide. In our experiment, administration of sodium selenite resulted in significant reduction of the activities of the antioxidant enzymes SOD, CAT, and GPx, whereas such a decline was prevented in the lenses of animals cotreated with saffron. It could be postulated that saffron improves the antioxidant defense mechanisms of the lens, leading to restoration of the levels of SOD, CAT, and GPx activities, in spite of exposure to sodium-selenite.

GSH, a tripeptide of glycine, glutamic acid, and cysteine, is found in unusually high levels in the crystalline lens and is believed to play a significant role in the maintenance of the reduced state in the lens. GSH (via its side sulfhydryl group) is involved in the detoxification of potentially damaging oxidants such as H_2_O_2_ and dehydroascorbic acid, and in the protection against oxidation of membrane –SH groups, which are important for cation transport regulation. Finally, it protects cytoskeletal proteins and prevents the crosslinking of soluble crystallins, and therefore, maintenance of its levels is vital for the preservation of lenticular transparency [43-45].GSH levels decrease with age and are found reduced in most types of cataract [45,46]. Furthermore, selenite overdose has been proved to cause a significant depletion of lens GSH levels [47,48]. Restoration of GSH and total glutathione levels could be attributed to the beneficial effects of saffron treatment on the enzymes involved in glutathione synthesis [49,50].

The crystallins are subject to oxidative changes, including the formation of disulfide and other inter- and intramolecular crosslinks, resulting in their aggregation [51]. Furthermore, one of the primary events in selenite-induced cataractogenesis theory is the oxidative damage that occurs to proteins of the lens epithelium membranes [39]. Free sulfhydryl (–SH) groups of proteins are mainly responsible for their antioxidant response, and they can serve as a sensitive indicator of oxidative stress [52]. Consequently, the reduced level of protein sulfhydryl content is an indication of tissue protein oxidation. In the present study, in contrast to the lower level of lens protein sulfhydryl content of the selenite-treated group, saffron coadministration kept the protein sulfhydryl content near to control levels, indicating its protective effect against oxidative damage.

Free radical–induced lipid peroxidation is a highly destructive process and has been implicated as one of the main components of the pathogenetic mechanism of selenite-induced cataractogenesis [47]. The accumulated peroxidation products damage vital membrane structures [53]. MDA is a product of the breakdown of mainly unsaturated fatty acids through the oxidation mechanism [54]. In the present study, such a disturbance of membrane lipids from selenite probably resulted in the observed increase in MDA lens levels when compared to normal lenses. The observed almost twofold reduction in the MDA level in the selenite+saffron-treated group suggests that saffron possibly preserved the structural integrity of the lens, thereby preventing its opacification.

Selenite cataract is characterized by a marked decline in water-soluble proteins through protein insolubilization either by excessive proteolysis by calpains or through structural alterations resulting from sulfhydryl oxidation [40]. The selenite-induced alteration in the lens protein profile has been depicted through the calculation of the soluble/insoluble protein ratio in the selenite group in our experiment. Saffron supplementation prevented protein degradation and aggregation, resulting in less cataract formation. Previous studies showed that many of the soluble proteins in the approximate molecular weight ranges of 23–31 kDa are from β-crystallin, 19–20 kDa are from α-crystallin, and the smear of polypeptides from 21 to 23 kDa are γ-crystallins [55]. Saffron coadministration resulted in considerable preservation of the pattern of lens proteins with molecular size 19–21 kDa and 25–30 kDa, which probably denotes inhibition of crystalline proteolysis.

To conclude, in the present in vivo study, we showed for the first time in the literature that intraperitoneal administration of saffron extract could significantly protect against nuclear opacity formation in selenite-treated pups. This indicates that saffron components/metabolites can penetrate the blood-aqueous barrier and reach the aqueous humor. It has been recently shown that crocetin, the main crocin metabolite, is determined in cerebral tissue after intraperitoneal administration of saffron extract [56]. The extent of tissue damage caused by selenite and the protective effect of saffron were evaluated clinically and biochemically. The modulatory effect of saffron seems to be associated with a reduction of the intensity of lipid peroxidation and protein oxidation, maintenance of antioxidant status, and inhibition of proteolysis of the lens WSF. This study has focused on the scientific validation of saffron as an anticataract agent, suggesting that saffron (or its components) exhibits a promising anticataractogenic effect. However, it is important to emphasize that certain differences exist between human and selenite experimental cataract. This preliminary study is encouraging, but further research is required to determine whether a similar anticataractogenic potential can be demonstrated in humans.
